# Angiogenesis is present in experimental autoimmune encephalomyelitis and pro-angiogenic factors are increased in multiple sclerosis lesions

**DOI:** 10.1186/1742-2094-7-95

**Published:** 2010-12-22

**Authors:** Timothy J Seabrook, Amanda Littlewood-Evans, Volker Brinkmann, Bernadette Pöllinger, Christian Schnell, Peter C Hiestand

**Affiliations:** 1 Department of Autoimmunity, Transplantation and Inflammation, Novartis Institutes for Biomedical Research, Basel Switzerland; 2 Department of Oncology, Novartis Institutes for Biomedical Research, Basel Switzerland

## Abstract

**Background:**

Angiogenesis is a common finding in chronic inflammatory diseases; however, its role in multiple sclerosis (MS) is unclear. Central nervous system lesions from both MS and experimental autoimmune encephalomyelitis (EAE), the animal model of MS, contain T cells, macrophages and activated glia, which can produce pro-angiogenic factors. Previous EAE studies have demonstrated an increase in blood vessels, but differences between the different phases of disease have not been reported. Therefore we examined angiogenic promoting factors in MS and EAE lesions to determine if there were changes in blood vessel density at different stages of EAE.

**Methods:**

In this series of experiments we used a combination of vascular casting, VEGF ELISA and immunohistochemistry to examine angiogenesis in experimental autoimmune encephalomyelitis (EAE). Using immunohistochemistry we also examined chronic active MS lesions for angiogenic factors.

**Results:**

Vascular casting and histological examination of the spinal cord and brain of rats with EAE demonstrated that the density of patent blood vessels increased in the lumbar spinal cord during the relapse phase of the disease (p < 0.05). We found an increased expression of VEGF by inflammatory cells and a decrease in the recently described angiogenesis inhibitor meteorin. Examination of chronic active human MS tissues demonstrated glial expression of VEGF and glial and blood vessel expression of the pro-angiogenic receptor VEGFR2. There was a decreased expression of VEGFR1 in the lesions compared to normal white matter.

**Conclusions:**

These findings reveal that angiogenesis is intimately involved in the progression of EAE and may have a role in MS.

## Background

Multiple sclerosis (MS) is a putative autoimmune disease of the central nervous system (CNS) and is one of the most common neurological diseases of young adults [1,2]. The exact cause of MS is unclear but appears to be a complex interaction of genetic, environmental and perhaps infectious causes [3,4]. It is characterized by multifocal inflammatory lesions in the white matter composed of lymphocytes, macrophages and activated glia, that result in demyelination and axonal damage [5]. Most MS patients present with bouts of disease activity (relapses) separated by periods of low disease activity (remission). Over time accumulating damage results in irreversible neurological impairment.

Besides the well characterized inflammatory infiltrate, disturbances in the blood brain barrier (BBB) occur in both MS and the animal model, experimental autoimmune encephalomyelitis (EAE) [6-8]. The BBB becomes permeable to plasma proteins such as IgG, including antibodies specific for myelin, which may promote disease severity [9,10]. Using magnetic resonance imaging (MRI) with gadolinium enhancement, these disturbances can be seen and quantified in MS patients [11,12]. The appearance of perfusion deficits, detected by MRI, has been shown to precede overt BBB breakdown [13]. However, the role of the blood vessels in initiation, propagation and resolution of MS plaque formation is still unclear.

Angiogenesis is a prominent feature of several CNS diseases including brain tumors, epilepsy and stroke [6,14-16]. Evidence is accumulating that angiogenesis may have a role in the pathophysiology of MS and EAE, similar to that seen in chronic inflammatory diseases of peripheral organs [17]. In EAE, histological examination has demonstrated an increased density of blood vessels in areas of inflammation [18,19]. Vascular endothelial growth factor (VEGF) is also increased at inflammatory sites during EAE and MS and infusion of VEGF worsens clinical scores during EAE [20]. Indeed an injection of VEGF alone into the CNS of naive rats can induce inflammation and angiogenesis [21]. There is also an increase in serum VEGF in MS patients in relapse compared to healthy controls or MS patients in remission [22]. A recent paper by Holley and colleagues demonstrated an increase in blood vessel density in MS lesions compared to normal controls [23] and increased proliferation of endothelial cells within these blood vessels. Together these data suggest that angiogenesis is occurring in EAE and MS.

The current experiments were performed to conclusively demonstrate angiogenesis during different disease phases of EAE, using vascular casting and histological techniques. To examine autoimmune induced neuroinflammation, we used the spinal cord homogenate induced EAE model in Lewis rats [24]. This model exhibits acute monophasic disease and spinal cord inflammation with subsequent relapse. This enabled us to analyze early acute (day 9-15) and relapse (day 21-27) phases of this disease. Furthermore, we examined MS lesions for changes in angiogenic factors, which may be responsible for driving the increased angiogenesis.

## Methods

### Induction and assessment of EAE

EAE is induced in female Lewis rats by guinea pig spinal cord homogenate plus complete Freund's adjuvant. Clinical symptoms present with an acute phase beginning at day 9 post immunization. This is followed by a remission period from days 15 to 20 and then a weaker relapse compared to the acute phase beginning at day 21 (average clinical score of 1 vs. 2.5 in the acute phase). The relapse is completed by day 28 in the majority of rats, with complete recovery at that time. 6-8 week old female Lewis rats (Harlan B.V., Horst, The Netherlands) were injected in the hind paws with 0.1 ml of an emulsion containing 25 mg of guinea pig spinal cord in complete Freund's adjuvant. Clinical grades of EAE were assessed using a clinical scale from 0 to 3. 0, normal, 1, loss of tail tonicity, 2, weakness of one or both hind legs, or mild ataxia and 3, severe ataxia or paralysis accompanied by urinary incontinence. In the current set of experiments 84% of the immunized rats achieved a relapse (defined as at least 2 consecutive days of a score equal to or greater than 1).

All animal experiments were approved by and performed in accordance to Novartis and local Swiss regulatory authorities.

### Vascular corrosion casts and quantification

Deeply anesthetized rats were thoracectomized and perfused through the left ventricle of the heart with rinsing solution (NaCl 0.9% with 250,000 U/l heparin at 35°C) at a controlled pressure of 120 mm Hg for 5 minutes at a rate of 20 ml/min. The right atrium was punctured to provide outflow. This was followed by a solution of 2% paraformaldehyde at 35°C, followed by up to 30 ml of polyurethane resin (PUII4; Vasqtec, Zürich, Switzerland) at the same rate. After 48 h, the resin-filled brain and spinal cord were excised from the animal and the soft tissue was removed by incubation with 7.5% KOH for 24 hr at 50°C. The casts were then thoroughly cleaned and stored in distilled water before drying by lyophilization.

Micro computer tomography measurements were performed with a desk-top MicroCT (μCT40, SCANCO Medical AG Bassersdorf, Switzerland). A voxel size of 8 μm in all three spatial dimensions was used. For each vascular cast, thoracic and lumbar slices were evaluated, covering a total of 20 mm. The determination of blood vessel size was performed with IPL software (SCANCO). An outer contour was produced automatically slice-by-slice, following a rough contour that had been drawn by hand in the starting slice. The region of interest (ROI) was defined within the contour. The grey-value images were segmented using fixed threshold to extract the blood vessels (threshold 1.5% of maximal grey-scale value which corresponds to a linear attenuation coefficient of 0.12 cm^-1^). The blood vessels were evaluated using the direct distance transformation method [25] to calculate their thickness.

### Histology

Deeply anesthetized rats were perfused with PBS followed by 4% paraformaldyde. Spinal cord and brain tissues were removed and postfixed in 4% paraformaldyde for 24 hours, followed by embedding in paraffin. Alternatively, following PBS perfusion, tissue was placed in OCT (Tissue-Tek) and snap frozen in dry ice cooled isopentane. Areas of the PBS perfused spinal cord were snap frozen for VEGF ELISA and stored at -80°C until required.

Two or three separate areas of the cervical, thoracic and lumbar area were examined from each rat. Sections from frozen (10 μm) and paraffin (7 μm) embedded tissues were mounted on slides and immunohistochemistry or histochemical staining was performed.

Paraffin sections were dewaxed, hydrated and antigen retrieval performed by heating sections at 98°C in citrate buffer pH6.0. Frozen sections were fixed in acetone for 10 minutes at room temperature. Immunohistochemistry was performed as previously reported [26]. A list of the primary antibodies and sources can be found in Table 1.

**Table 1:** Antibodies used for immunohistochemistry

Primary Antibody	Source	Isotype	Species Reactivity*	Clone or Reference	Dilution	Supplier
Angiopoietin-1	Mouse monoclonal	IgG1	h, m, r	F-1	4 μg/ml	Santa Cruz Biotechnology, Santa Cruz, CA
Angiopoietin-1	Rabbit polyclonal	IgG	h, m, r	Ab65835	2 μg/ml	Abcam, Oxford, UK
GFAP	Rabbit polyclonal	IgG	h, m, r	G560A	1 μg/ml	Promega, Madison, WA
Meteorin	Rabbit polyclonal	IgG	h, m	Ab56129	2 μg/ml	Abcam
Meteorin	Goat polyclonal	IgG	m	AF3475	6 μg/ml	R&D Systems, Abington, UK
Tie-2	Rabbit polyclonal	IgG	h, m, r	Sc-9026	2 μg/ml	Santa Cruz
VEGF	Rabbit polyclonal	IgG	h, m, r	sc-152	1 μg/ml	Santa Cruz
VEGF	Mouse monoclonal	IgG1	h, m, r	VG-1	3 μg/ml	Abcam
VEGFR1 (Flt-1)	Rabbit polyclonal	IgG	h	Sc-74007	2 μg/ml	Santa Cruz
VEGFR1	Rabbit polyclonal	IgG	h, m, r	Ab2350	1 μg/ml	Abcam
VEGFR2 (Flk-1)	Rabbit polyclonal	IgG	h, m, r	Ab2349	3 μg/ml	Abcam
VEGFR2	Rabbit polyclonal	IgG	h, m, r	Sc-504	2 μg/ml	Santa Cruz
Collagen IV	Rabbit polyclonal	IgG	r, m	Ab19808	2 μg/ml	Abcam
CD31	Rabbit polyclonal	IgG	h, m	Ab78364	2 μg/ml	Abcam

* h = human, m = mouse, r = rat

Myelin was visualized by immersing slides in solochrome cyanine RS (0.2%) for 10 minutes, washing in tap water for 5 minutes and differentiating in iron alum (10%) until the white and grey matter could be differentiated.

Frozen human control white matter and white matter containing chronic active lesion was obtained from the Netherlands Brain Bank (see Table 2 for subject details). Cryosections (10 μm) were thawed onto slides, dried for 30 minutes and stored at -80°C until required. Sections were fixed in acetone for 5 minutes, endogenous peroxidases quenched using 0.3% hydrogen peroxide in PBS for 15 minutes and blocked in 10% normal human AB serum for 30 minutes. The diluted primary antibody was added for 60 minutes and visualized with the Envision biotin free kit (DakoCytomation, Glostrup, DK). Lesions were confirmed to be chronic active by an area of well demarcated demyelination surrounded by HLA+ macrophages and gliosis.

**Table 2:** Subject information for CNS tissue used in immunohistochemistry

Case number	sex	age	Post-mortem delay (h)	Cause of death
MS1	F	41	8.25	natural causes
MS2	F	50	7.45	euthanasia
MS3	F	53	10.45	euthanasia
MS4	F	78	11.10	cerebral vascular accident
MS5	M	43	8.30	pneumonia
MS6	M	53	5.30	respiratory insufficiency
MS7	M	56	8.0	pneumonia
MS8	F	66	8.30	euthanasia
MS9	F	66	6.0	unknown
MS10	F	44	11.10	CVA relapse

Average ± SD		55 ± 11.9	8.4 ± 2	
				
C1	F	68	5.45	euthanasia (cancer)
C2	F	78	4.15	cerebral vascular accident
C3	M	48	5.30	euthanasia
C4	M	53	14.25	heart failure
C5	M	56	9.15	myocardial infarct

Average ± SD		60.6 ± 12	7.7 ± 4	

Normal rabbit or mouse IgG was used as a negative control and found to be negative in all cases.

### Quantification of histology

Solochrome, rat IgG, VEGF, VEGFR1, VEGFR2, Tie-2, angiopoientin-1 and meteorin stained sections were examined and the staining pattern determined. In many instances two different antibodies were used to stain the tissues. Although each pair of antibodies stained with different intensities, they recognized the same cells and/or structures.

For human tissue the staining pattern for each protein of interest was examined in the center, active rim and surrounding normal appearing white matter in the MS subjects (n = 12 lesions from 10 separate individuals). This was compared to white matter from normal control subjects (n = 5). Whenever possible, 2 different antibodies were used to confirm the staining pattern for each antigen.

For blood vessel density, rat spinal cord tissue sections stained for CD31 and collagen IV were examined. Using a 40× objective, the number of blood vessels in the grey matter (dorsal and ventral horns) and white matter (dorsal, lateral and ventral) were counted in each field. The resulting 4 values for the grey and white matter were averaged for each section. For each rat 2-5 separate areas of the cervical, thoracic and lumbar spinal cord were counted and an average calculated for each rat. The spinal cord segments were identified by counting the vertebrae during the tissue collection.

### ELISA

The spinal cord was removed from PBS perfused rats, separated into lumbar, thoracic and cervical areas, and snap frozen in liquid nitrogen. Tissue homogenates were made in PBS containing protease inhibitors, the supernatant collected after centrifugation and stored at -80°C until required. A specific rat VEGF ELISA kit (R&D systems Abington, UK) was used to measure VEGF levels.

### Statistical analysis

An unpaired Student's T-test was carried out to determine significance (GraphPad Prism version 4.00 for Windows (GraphPad Software, San Diego California USA). A value of p < 0.05 was considered significant.

## Results

### Blood vessel density increases during EAE

**Figure 1:**
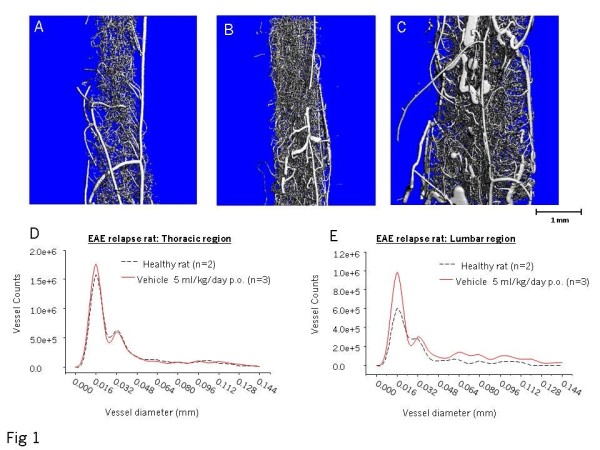
**There is an increase in small blood vessels in the relapse phase of EAE**. Vascular casting shows the vascular pattern of the lumbar spinal cord in the normal rat (A). During acute EAE (B) there is no significant increase in blood vessel numbers. However, in the relapse phase (C) there is an increase of blood vessel numbers, which shows increased tortuosity compared to healthy rats. Quantification using μCT demonstrates that in the thoracic region of the spinal cord during EAE relapse there is no significant increase in blood vessel numbers (D). In contrast, in the lumbar region versus the thoracic region, there is a significant increase in small diameter vessels (all vessels smaller than 24 μm) Student T test (p = 0.011) (E).

To investigate whether angiogenesis is present during EAE, vascular casting was performed during the acute (day 14) and relapse (day 27) phase of disease and compared to those obtained from naive rats. Visual inspection of the casts showed an increase in blood vessels during the relapse phase but not during the acute period of disease (Figure 1). These new blood vessels appear to be more torturous, with increased size, dilations and curvatures in EAE rats compared to those found in naive rats. The grey matter is located in the center of the cast and in healthy rats it is known that this area contains a greater number of blood vessels [27]. By visual inspection during EAE relapse, the increase in the number of blood vessels in the grey matter appeared to be greater than in the peripheral white matter, though the white matter also had an increase in blood vessel density. In this Lewis rat EAE model, inflammation is found in both the grey and white matter due to immunization with spinal cord homogenate. In addition, we constantly observe an inflammatory gradient with the lumbar region being more severely affected than the thoracic and cervical regions in turn. This is due to the fact that this model exhibits an ascending spinal cord paralysis (data not shown). A difference in edema and resultant swelling in the different regions therefore will also impact on blood vessel counts.

To quantify the vascular casts, μCT was performed. Confirming the visual examination, there was a significant increase in blood vessel numbers in the lumbar region of the spinal cord but not the thoracic spinal cord (compare Figure 1D and 1E). The increase in blood vessel numbers was most prominent in the small diameter blood vessels (24 μM in diameter) but there was an increased number in all diameters including those over 0.1 mm.

**Figure 2:**
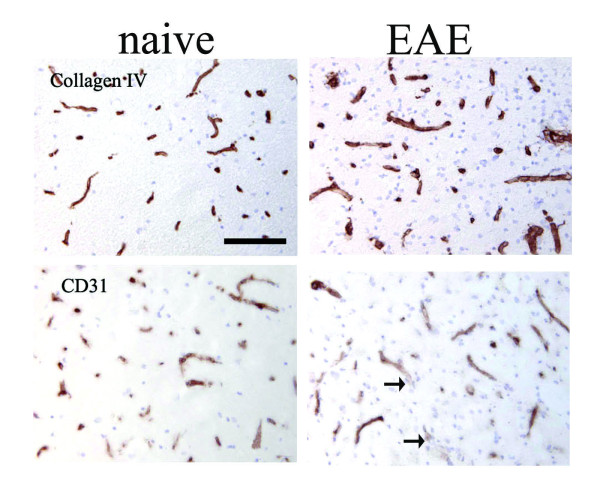
**Increased blood vessel density as seen with collagen IV and CD31 immunohistochemistry**. Representative collagen type IV and CD31 immunohistochemical stains from the lumbar spinal cord region. In the white matter of the naïve rats, collagen IV and CD31 blood vessels are found. In the EAE rats during the relapse phase there are more collagen IV positive blood vessels seen in the inflamed white matter. CD31 immunohistochemistry shows a similar increase in blood vessel density. Interestingly in some of the blood vessels, the CD31 staining became much more diffuse (arrows) and not as prominent as compared to the naïve rats.

Inflammation induced vasodilatation of small blood vessels may allow better perfusion by the casting material, thus erroneously showing an apparent increase in vessel density. To determine whether this was the case, a separate experiment was performed and histology was used to quantify the number of blood vessels in the cervical, thoracic and lumbar spinal cord of naive rats and during the relapse phase of EAE (day 25). Immunohistochemistry using antibodies to CD31 and collagen IV was used to label blood vessels and counting was performed on spinal cord white and grey matter. Collagen IV staining demonstrated a significant increase in blood vessel density during the relapse phase in the grey (p < 0.01) and white (p < 0.001) matter of the lumbar spinal cord and in the thoracic white and grey matter (p < 0.05) as compared to naive rats (Table 3). To confirm these data, CD31 expressing blood vessels were counted. These results were similar to those found for collagen IV (Table 3). Similar to previous results we found that CD31 staining was present in a more diffuse pattern on endothelium in the inflamed CNS on some blood vessels (Figure 2) [28] and even disappeared on other blood vessels as noted by Tham and colleagues [29].

**Table 3:** Increased blood vessels during EAE

		CD31		Collagen IV	
		
		naive	EAE	naive	EAE
		
Lumbar	WM	3.1 ± 0.1*	7.7 ± 1.2^†^	2.8 ± 0.5**	8.7 ± 1.2
	GM	6.7 ± 0.4 **	13.0 ± 1.4	8.8 ± 1.6**	16 ± 1.5
Thoracic	WM	2.9 ± 0.2**	5.9 ± 0.6	3.1 ± 0.2**	6.9 ± 0.6
	GM	6.5 ± 0.7**	13 ± 1.2	9.2 ± 1.3*	17 ± 1.8

* p < 0.05 compared to EAE

**p < 0.01 compared to EAE

^†^Number of vessels counted using a 40× microscope objective

Blood vessel density was higher in the grey matter than in the white matter, similar to that seen in the vascular casting. However, unlike the vascular casting, histological examination also demonstrated an increase in vessel density within the thoracic spinal cord. An explanation for this discrepancy is that vascular casting depends on the blood vessel being patent. Histological examination will detect all blood vessels including those that are newly formed and not perfusable. Nevertheless, using two different methods we found an increase in blood vessel density during EAE.

**Figure 3:**
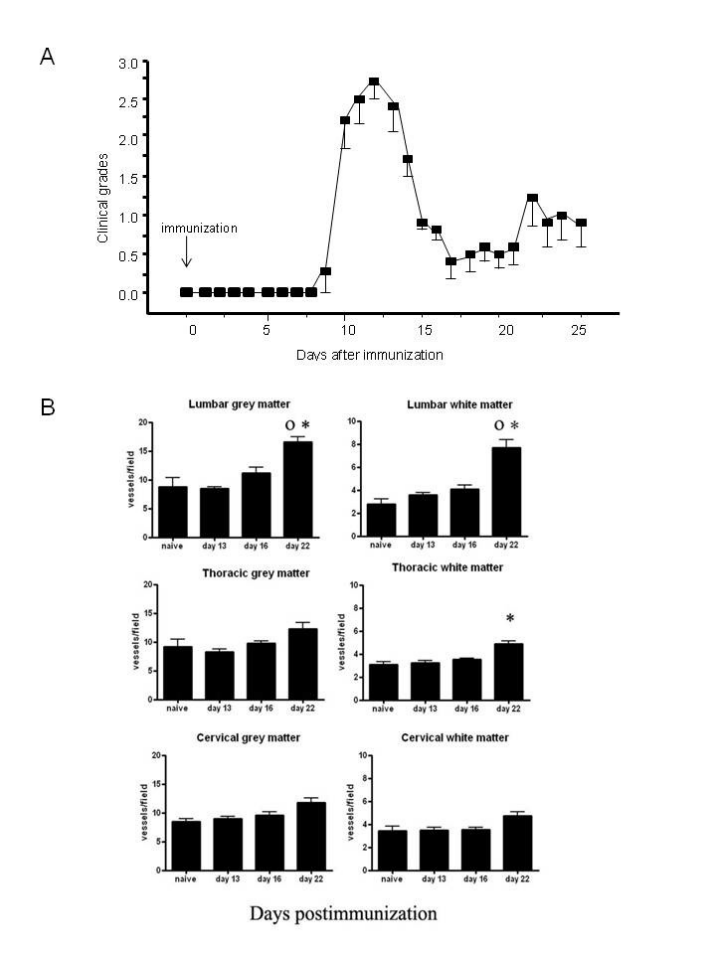
**Blood vessel counts are increased in both the grey and white matter during EAE relapse**. (A) Clinical grade of animals during the different phases of EAE. Animals display neurological impairment during the acute phase and relapse, whereas they show almost complete remission between the two episodes. The acute inflammatory phase ranges from 9 to 17 dpi, the remitting phase from 17 and 21 dpi and relapse from 21 to 26 dpi. (B) Collagen IV immunohistochemistry and subsequent quantification shows a significant increase of blood vessels at day 22 (relapse) in the lumbar spinal cord grey and white matter. There is little change in blood vessel numbers in either the thoracic or cervical spinal cord, with the exception of a small increase in the thoracic white matter at day 22 post immunization. n = 5/rats timepoint mean ± S.D. ° p < 0.05 compared to day 13, * p < 0.05 compared to naive rats.

We then investigated if the blood vessel density changed during the different phases of EAE. Tissue was harvested from rats at different times after immunization and blood vessels were quantified using collagen IV staining. The number of blood vessels increased during the relapse phase in the grey (p < 0.01) and white (p < 0.001) matter of the lumbar spinal cord and in the thoracic white matter (p < 0.05) as compared to naive rats (Figure 3). There was a slight, but insignificant increase at day 16 in the lumbar spinal cord grey matter, suggesting that at this phase, blood vessel numbers began to increase. We did not examine the tissues for CD31 as this marker was reduced on the blood vessels of some EAE animals which made counting more difficult.

### Angiogenesis mediators are modulated during EAE

**Figure 4:**
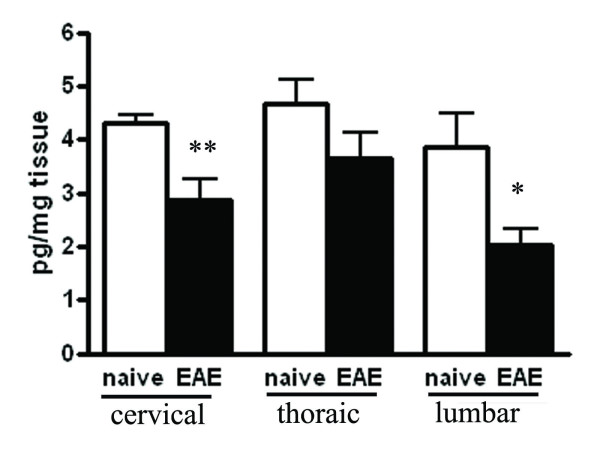
**EAE results in a significant decrease in spinal cord VEGF**. Spinal cords were harvested during the relapse phase of EAE, divided into cervical, thoracic and lumbar areas, homogenized and measured using a specific rat VEGF ELISA kit. There is a significant decrease in VEGF in the cervical and lumbar areas in EAE rats compared to naïve controls. * p < 0.05, **p < 0.01 n = 5 rats per group

VEGF has been shown to be expressed in MS [30] and is a potent inducer of angiogenesis in the brain, therefore we determined whether this pro-angiogenic growth factor was increased during the relapse phase of EAE. Cervical, thoracic and lumbar areas of the spinal cord isolated from naive and EAE rats following PBS perfusion (n = 5/group) were measured using a VEGF specific ELISA. In both the cervical and lumbar areas of the spinal cord the rats with EAE had significantly lower VEGF levels compared to the naïve rats (Figure 4). In the thoracic spinal cord the EAE rats had a lower VEGF level but it did not reach significance.

**Figure 5:**
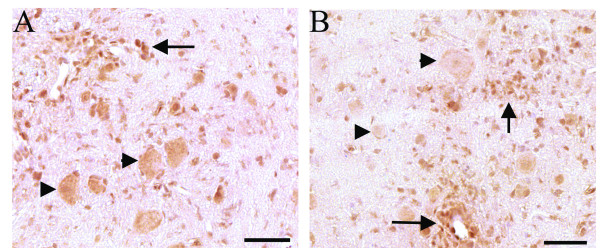
**Inflammatory infiltrates express VEGF but neuronal expression is decreased during EAE relapse**. In acute EAE some cells in the inflammatory infiltrate (arrow) and spinal cord neurons (arrowheads) express VEGF (A). During EAE relapse there is a decrease in neuronal expression of VEGF (arrowheads) but some infiltrating cells near blood vessels continue to express VEGF (arrows, B). Scale bar = 100 μm

To investigate this decrease, we performed immunohistochemistry for VEGF at all stages of EAE. We found that during the acute and relapse phases of EAE there were VEGF immunopositive cells in the perivascular inflammatory infiltrate (Figure 5). Additionally, VEGF positive neuronal cell bodies were found throughout the spinal cord in both naive rats and during acute EAE. During the relapse phase of EAE, there were less VEGF immunopositive neurons (Figure 5B). This decrease in VEGF positivity in the neurons is likely responsible for the observed decrease in VEGF measured by ELISA, even in the presence of VEGF positive inflammatory infiltrates.

**Figure 6:**
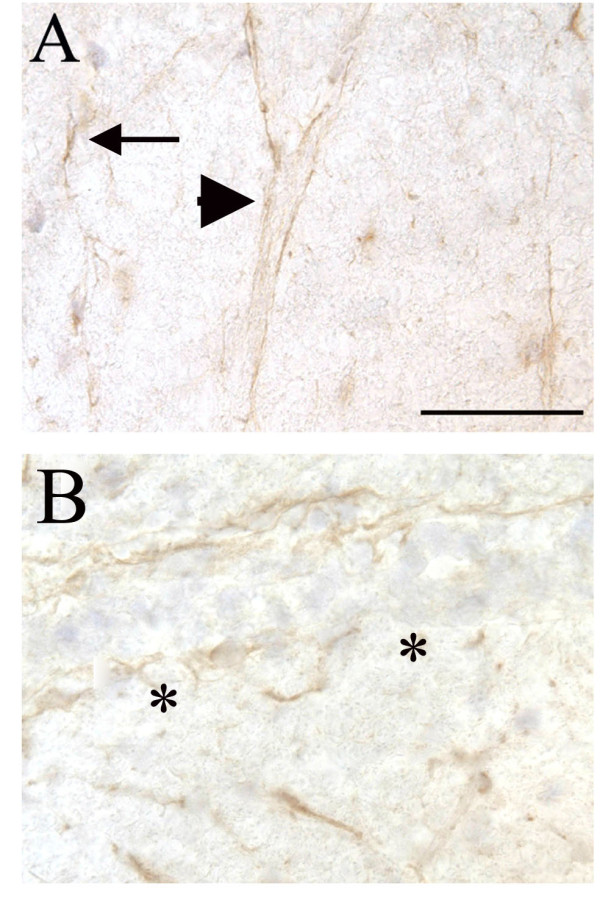
**Vascular meteorin expression is affected during EAE in the lumbar spinal cord**. Meteorin immunoreactivity is closely associated with many blood vessels and appears to completely surround blood vessels in naive rats (A, arrowhead). In addition there are a few branched cells in the parenchyma expressing meteorin, which resemble astrocytes (arrows, A). In relapsing EAE rats there are breaks in the blood vessel (asterisks, B) associated meteorin in the white matter of the lumbar spinal cord. scale bar 50 μm.

Other mediators of angiogenesis in the brain during disease include Tie-2 (Tek), VEGFR2 (Flk-1) and meteorin. As these mediators have not been previously characterized in EAE, their expression was examined during all phases of disease. Meteorin is a recently described molecule that is secreted by astrocytes in vitro to attenuate angiogenesis and is located on astrocyte endfeet in the normal brain [31]. In agreement with previous reports, we found meteorin localized to the abluminal side of blood vessels (Figure 6). During the relapse phase of EAE we found a disrupted expression of meteorin immunoreactivity along some inflamed blood vessels. Spinal cord tissue harvested from acute phase rats had occasional VEGFR2 positive blood vessels, the expression of which did not change during EAE (data not shown). We did not detect expression of VEGFR1 or Tie2 by endothelial cells or other cells during any of the phases of EAE. This may be due to the insensitivity of the antibodies available for these antigens in rats (data not shown).

### Angiogenesis mediators are changed in multiple sclerosis lesions

**Figure 7:**
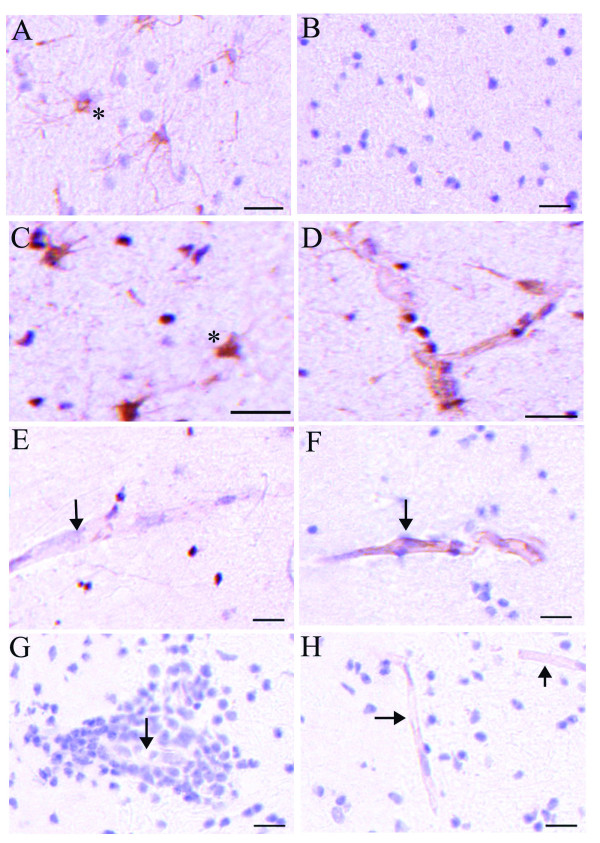
**Increased VEGF and VEGFR2 but decreased VEGFR1 in MS lesions**. Glial cells expressed VEGF in the rim of chronic demyelinated lesions in > 70% of the samples (asterisks, A) but there was no expression in the white matter of any of 5 control subjects (B). Glial cells (asterisks, C) and blood vessels (D) in areas of demyelination located in the center of the lesion and the rim expressed VEGFR2 in > 70% of samples, but there was minimal staining in all the normal subjects (E, arrow points to blood vessel). Blood vessels in all normal subjects expressed VEGFR1 (arrow, F) but there was a reduction in blood vessels surrounded by inflammation (arrow, G) or in the central area of the lesion (arrow, H) in the majority of subjects with MS. scale bars = 20 μm.

To observe whether angiogenesis is a component of MS, we next examined active chronic MS lesions for changes in angiogenic proteins. Glial cells in the rim of the active lesion expressed VEGF (Figure 7A), whilst in normal subjects VEGF expression was not detected by immunohistochemistry (Figure 7B). Many glial cells in the rim of the active chronic lesion and some blood vessels expressed VEGFR2 (Figure 7C, D). There was minimal expression of VEGFR2 in white matter from control subjects (Figure 7E). The angiogenic mediator, VEGFR1 (Flt-1) was detected on the majority of blood vessels in the control subjects (Figure 7F) and in the normal appearing white matter of MS subjects (not shown). Blood vessels surrounded by perivascular infiltrates (Figure 7G) or in central areas of the demyelinated lesion (Figure 7H) expressed less VEGFR1 in greater than 70% of the MS subjects, as compared to those found in control brains. Tie2 was found on the majority of blood vessels in both control white matter and throughout the MS lesions (data not shown). There was no staining for the Tie2 ligand angiopoietin-2, in either the control white matter or MS lesions. Meteorin was found to be expressed on some blood vessels in both control white matter and in some MS subjects but there was no clear change in expression in relation to MS lesion location (data not shown).

## Discussion

These data demonstrate that angiogenesis occurs during EAE similar to that seen in other chronic inflammatory diseases [32]. Previously angiogenesis was suggested by increased plasma VEGF levels [22] and increased blood vessel density in EAE [18,19]. Using vascular casting and histology, we demonstrate an increase in blood vessel density specifically during the relapse phase, but not during the acute phase of EAE. In particular, we observed that angiogenesis prevailed in the lumbar region which most likely is in line with the ascending paralysis that is observed with this model. However, a more detailed analysis of the angiogenic potential of specific infiltrating immune cell subtypes and the response by neural cells needs to be performed.

Chronic inflammatory diseases induce angiogenesis by a variety of mechanisms. Macrophages and T cells produce cytokines such as TNF, IL-8, TGF-β and MMP-9, which are known angiogenic factors [33,34]. Furthermore macrophages can produce VEGF, the most potent known inducer of angiogenesis [35,36]. As these same cells and soluble factors are found in EAE and MS lesions it is perhaps not surprising that angiogenesis is occurring [37]. Indeed the main location of inflammation is the perivascular area of the CNS [5], which is ideal for acting upon endothelial cells and smooth muscle cells to induce angiogenesis. Our current data demonstrates that new blood vessels are formed only during the relapse phase of EAE. This suggests that the vasculature does not respond quickly to neuroinflammation but must be acted upon in a more chronic manner. Further experiments examining a more chronic EAE model such as myelin oligodendrocyte glycoprotein induced EAE in the DA rat model would address this aspect. In addition the cellular infiltrate differs over time, which may change the angiogenic milieu within the CNS, thus the effects of the different inflammatory subtypes on glia, neurons and the BBB should be examined. Lastly the difference between the lumbar and thoracic spinal cord are interesting and may point to differences in the physiology of these two anatomical locations. Alternatively, the differences may be due to the inflammation being more intense and/or chronic in the lumbar. The vascular casting demonstrated that the increase was seen in small blood vessels, whilst the larger vessel numbers remained stable. This finding is logical as it would be expected that the newly formed blood vessels would be smaller. Our findings are somewhat different to those recently reported by Roscoe and colleagues [19], as they found an increase in laminin positive blood vessels during the acute phase of EAE. This could be due to a number of factors including species differences (mouse vs. rat, current study) and method of EAE induction (MOG_35-55 _plus pertussis toxin vs. spinal cord homogenate, current study). Nonetheless, both studies reported an increase in blood vessels during EAE. Therefore, using both histological examination and vascular casting we have demonstrated that angiogenesis occurs in EAE and thus may also be present in MS.

Angiogenesis in the adult brain is a complex interaction of several pathways involving various different receptors and signaling molecules [6,38]. Mueller et al. recently published an extensive gene array analysis of myelin oligodendrocyte protein induced EAE in the DA rat [39]. Examination of this data set showed modulation of several angiogenic factors including VEGF and its receptors. Many of these same proteins are implicated in angiogenesis found in tumours, stroke and epilepsy [14-16]. Based on these data we examined VEGF, VEGFR1, VEGFR2, Tie-2, angiopoietin-1 and meteorin in the current study. During the relapse phase of EAE meteorin expression was discontinuous along some blood vessels. Meteorin is located on astrocytic endfeet and acts through thrombospondin-1/2 to promote vasculature maturation [30]. As far as we are aware, this is the first report that meteorin expression is changed during neuroinflammation. In human MS lesions, there was an increase of VEGFR2 expression by both glial cells in the rim of the lesion and blood vessels. There was decreased VEGFR1 expression by blood vessels that had a perivascular cuff of inflammation and those located in demyelinated areas. As previously reported, Tie-2 was present on CNS blood vessels but we did not find angiopoietin-1 expressed. These data demonstrate that a pro-angiogenic environment is present in the inflamed brain.

The expression of VEGF during EAE is controversial, with both increased [19,20] and decreased [29] expression reported. We found a decreased level of VEGF as measured by ELISA. Immunohistochemistry demonstrated that neuronal expression of VEGF was present during acute EAE, whilst during the relapse phase, neuronal VEGF was decreased. Our data suggest that the contradictory results reported are due to a number of differences. Both Tham et al [29] and Proescholdt et al [20] used guinea pig MBP peptide induced EAE in Lewis rats. Firstly, the timing of the tissue analysis is important, as we have found that VEGF is expressed by neurons and inflammatory infiltrates in acute EAE but neurons decrease expression during relapse. Secondly, the spinal cord area examined will influence the results. Proescholdt and colleagues examined the white matter, whilst Tham and colleagues examined neurons in the grey matter and lumbar spinal cord homogenate. As we found VEGF to be expressed by infiltrates but decreased in neurons after the acute phase of disease, this may explain some of the differences. Lastly, there are technical differences due to different antibodies used, immunohistochemical protocols and detection methods (in situ hybridization, RT-PCR and ELISAs). Nevertheless, our data demonstrates that VEGF is modulated during EAE and may have a role in its pathophysiology. Further experiments using double immunohistochemistry and flow cytometry should be performed to examine the production of angiogenic factors by the different inflammatory cells (Th1, Th17, Tregs, macrophages ect.) and determine changes over the course of EAE.

The downregulation of VEGF in the neurons during relapse could be due to a number of mechanisms. As VEGF has a role in neuroprotection [40], its decrease by neurons may be involved in neural damage seen in the paralytic episodes following the acute phase. McCloskey and colleagues have demonstrated a decrease in neuronal VEGF following excitation induced by convulsions [41], therefore perhaps a similar mechanism exists during EAE. Alternatively, since it has been demonstrated that VEGF-A can disrupt the BBB, the decrease in neuronal VEGF may act as a counter regulatory mechanism to balance the increased VEGF due to the inflammatory infiltrates [42]. Lastly, the inflammation may supply exogenous VEGF to the neurons, thus allowing neurons to reduce endogenous VEGF production.

In the MS lesions, glial cells in the active rim of the lesion expressed VEGF, similar to that reported by others [20,43]. Therefore, both the receptor VEGFR2 and its ligand VEGF are expressed in the MS lesion. The expression of VEGFR2 by endothelial cells likely has an important role in the formation of new blood vessels, while the role on astrocytes is less clear. In humans there have been reports of astrocytoma expression of VEGFR2 [44] therefore a similar pathway may be activated in MS lesions to promote angiogenesis and glial survival or activation.

Overall, these data demonstrate that angiogenesis is increased in EAE and that a pro-angiogenic environment exists in MS lesions. Angiogenesis is increased during the relapse phase of EAE and therefore may be central to the propagation of the chronic immune response seen in MS.

## Competing interests

TS, BP, VB, CS, ALE and PH are or were employees of Novartis Institutes for BioMedical Research and/or own Novartis shares. The authors declare that they have no competing interests.

## Authors' contributions

TS performed the immunohistochemical procedures, interpreted these data and drafted the manuscript. A L-E contributed to the data interpretation and drafted the manuscript. BP contributed to the data interpretation and reviewed the manuscript. VB contributed to data interpretation. CS performed the vascular casting experiments and interpreted these data. PH supervised the EAE experiments, analyzed data and initiated the project. All authors read and approved the final manuscript.
